# Docosapentaenoic Acid (DPA, 22:5*n*-3) Alleviates Ulcerative Colitis via Modification of Gut Microbiota and Their Metabolism

**DOI:** 10.3390/nu14194204

**Published:** 2022-10-09

**Authors:** Ye Dong, Cheng Huang, Jiacheng Yang, Zhenxiao Zheng, Zhiyuan Dai

**Affiliations:** 1 Collaborative Innovation Center of Seafood Deep Processing, Zhejiang Province Joint Key Laboratory of Aquatic Products Processing, Institute of Seafood, Zhejiang Gongshang University, Hangzhou 310012, China; 2 Greentown Agricultural Testing Technology Co., Ltd., Hangzhou 310052, China

**Keywords:** docosapentaenoic acid, ulcerative colitis, gut microbiota, fecal metabolites

## Abstract

*N*-3 polyunsaturated fatty acids (*n*-3PUFA) are regarded as viable alternatives to aid the treatment of ulcerative colitis (UC). Most research focuses on eicosapentaenoic acid (EPA) or docosahexaenoic acid (DHA); little information is available about the effect of docosapentaenoic acid (DPA) on the gut microbiota and their metabolism in UC mice. In this study, the changes in gut microbiota and their metabolism in UC mice were studied through the 16S rRNA sequencing method and untargeted metabolomics. Moreover, the differential bacterial genus and differential metabolites in responding to DPA supplementation were screened through permutation test after orthogonal partial least squares discriminant analysis (OPLS-DA). The results indicated that DPA supplementation increased the diversity and altered the composition of the gut microbiota in UC mice; *Akkermansia*, *Alistipes*, *Butyricicoccus*, and *Lactobacillus* were selected as the differential bacterial genus. Supplementation of DPA also altered the fecal metabolite profile in the UC mice. Moreover, butyrate, *N*-carbamylglutamate (NCG), and histamine were screened as the differential metabolites. In conclusion, the regulation effect of DPA on the gut microbiota and their metabolism might be involved in the intervention mechanism of DPA in UC. More research needs to be carried out to elucidate the mechanism systematically.

## 1. Introduction

Ulcerative colitis (UC) is a nonsexual chronic inflammatory intestinal disease with diarrhea, abdominal pain, and hematochezia [1]. In recent years, the incidence rate of UC has been increasing continuously. The incidence rate of UC in North Europe is the highest (57.9/100,000), followed by North America (23.14/100,000), East Asia (4.6/100,000) and Africa (3.29/100,000) [2]. Since the principal patients of UC are young people and it is difficult to cure, the course of UC could last for decades. Therefore, UC not only brings great pain to the patients, but greatly affects social productivity. Based on these findings, UC is regarded as one of the most challenging diseases of modern times by the WHO [3,4]. At present, an aminosalicylic acid preparation, antibiotics, immunosuppressants, and glucocorticoid are common medicines to relieve UC. Although these drugs can alleviate the symptoms of UC, their side effects, and drug resistance, cannot be ignored. In view of the close relationship between the occurrence of UC and dietary factors, dietary intervention has become a promising research field. Among dietary factors, fatty acids play an important role. Studies have confirmed that the risk of UC in people with sufficient *n*-3PUFA intake is significantly lower than that in people with insufficient *n*-3PUFA intake. Moreover, increasing the proportion of *n*-3PUFA in a patient’s diet could also relieve the clinical symptoms [5,6,7].

However, most research focuses on *n*-3PUFA mixture, EPA, or DHA; DPA has received little attention. Wang et al. [8] intervened in UC mice with mussel lipid extract rich in *n*-3PUFA, and the results showed that *n*-3PUFA mixture significantly alleviates the symptoms of UC in mice by inhibiting the TLR_4_/MAPKs/NF-κB inflammatory signaling pathways. Liu et al. [9] studied the intervention mechanism of *n*-3PUFA mixture, extracted from Antarctic krill oil, in UC from the perspective of gut microbiota. The results indicated that *n*-3PUFA relieved UC by improving gut microbial dysbiosis. Another epidemiological study showed that DHA supplementation significantly increased the diversity of intestinal flora and improved the dysbiosis of amino acid metabolism in colitis patients [10]. The chemical structure and metabolites of DPA are different from those of EPA and DHA. Moreover, research has indicated that DPA has different pharmaceutical effects to EPA and DHA [11]. It has been reported that DPA possesses 10-fold greater endothelial cell migration ability than EPA [12]. An in vivo study reported that DPA reduced the fatty acid synthase and malic enzyme activity levels in mice and these effects were stronger than other *n*-3PUFA [13]. Our previous study [14] indicated that DPA significantly inhibited bodyweight loss and colonic shortening of dextran sulphate sodium (DSS)-induced mice and we explained the effect from the perspective of inflammatory response preliminarily. In recent years, with the deep realization of gut microbiota, more and more evidence has indicated that the effect of *n*-3PUFA on gut microbiota plays an important role in their intervention mechanism in UC. Therefore, we supposed that DPA was helpful in the regulation of the dysbiosis of gut microbiota composition and their metabolites.

In this study, the UC model was established through the DSS-induced C57BL/6 mice. Body weight decrease, disease activity index (DAI) score, and colon morphology were used to evaluate the protective effect of DPA on UC. Then, the effect of DPA on the gut microbiota in the UC mice was studied through the 16S rRNA sequencing method. The classification and diversity of the gut microbiota were analyzed by the bioinformatics platform and the differential bacterial genus of DPA supplementation was screened through permutation test after orthogonal partial least squares-discriminant analysis (OPLS-DA). Next, the effect of DPA on the fecal metabolites in the UC mice was studied through untargeted metabolomics. The correlation analysis of the metabolites in different groups was conducted by principal component analysis (PCA) and the differential metabolites between different groups were screened through permutation test after OPLS-DA. Finally, the interplay of the differential bacterial genus, differential metabolites, and UC was discussed. We hope this work can expand the biological effects of DPA and provide scientific support for dietary control of UC.

## 2. Materials and Methods

### 2.1. Materials and Animals

High purity (>97%) DPA, EPA, and DHA ethyl esters were prepared through distillation, silver ion complexation, and dynamic axial compression chromatography in our laboratory. Dextran sulfate (mw36000-50000): Mp biomedicals, USA. 4% polyformaldehyde general tissue fixation liquid: Solebao, China. Male C57BL/6 mice (8 weeks old) were purchased from the Centre of Laboratory Animals, Zhejiang Academy of Medical Sciences. Mice were housed under standard conditions (26 ± 1 °C, 50% ± 5% humidity and 12 h light/dark cycle). Before the experiment, mice were acclimatized for one week. The Animal Ethics Committee of Zhejiang Gongshang University (21211301) approved the experimental protocols in this experiment.

### 2.2. Grouping and Modeling

A total of 50 mice were randomly divided into 5 groups: EPA group, DPA group, DHA group, model control (MC) group, and health control (HC) group. Groups EPA, DPA and DHA were given 300 mg/kg/day of EPA, DPA, and DHA (dissolved in olive oil) by gavage, and autoclaved water was replaced by 3% DSS solution during the fourth week. The MC group was given the same volume of olive oil during the experimental period, and autoclaved water was also replaced by 3% DSS solution during the fourth week. The HC group was given the same volume of olive oil during the experimental period and they were given autoclaved water during the experimental period.

### 2.3. Body Weight and Disease Activity Index (DAI) 

During the last week of the experiment, the body weight change rate (0–4 points), fecal consistency (0–4 points), and fecal occult blood (0–4 points) of mice in each group were monitored daily and scored. Fecal occult blood of mice was detected by fecal occult blood qualitative detection kit (TC0511, Beijing Regan Biotech Co., Ltd., Beijing, China) according to the instructions of the kit. The DAI score is the average score of the body weight change rate, fecal consistency and fecal occult blood.

### 2.4. Colon Length and Morphology 

After the experiment, the mice were euthanized. The mice were dissected to obtain their colon tissue (from the end of cecum to anus). The colon tissues were rinsed by phosphate buffer solution, and then they were placed on an ultra-clean workbench to measure the length and weight. Then, the feces in the colon were removed for the subsequent testing.

### 2.5. DNA Isolation, PCR and 16S rRNA Analysis

At the end of the experiment, the mice were killed and the colon was dissected. The fresh feces were treated with liquid nitrogen and crushed in a mortar. Genomic DNA of intestinal microorganisms in the fecal matter was extracted by the fecal genomic DNA extraction kit (Tiangen, DP328). The V3~V4 hypervariable region of microbial 16S rRNA was amplified by primers: 338F (5′-ACTCCTACGGGAGGCAGCAG-3′) and 806R (5′-GGACTACHVGGGTWTCTAAT-3) by a PCR termocycler (LC-Bio Technology Co., Ltd., Hangzhou, Zhejiang, China). The PCR reaction was conducted in the 25 μL system. The system contained PhusionHot start flex 2X Master Mix (12.5 μL), 338F (2.5 μL), 806R (2.5 μL), template DNA (50 ng), and PCR-grade water to adjust the volume. The PCR reaction procedure was as follows: (1) 98 °C, 30 s; (2) 98 °C, 10 s; (3) 54 °C, 30s; (4) 72 °C, 45 s; (5) 72 °C, 10 min; (6) 4 °C, ∞; (2) to (4) repeated 35 times. The PCR amplification products were detected by 1.5% agarose gel electrophoresis. The target fragments were recovered using the AxyPrep PCR Cleanup Kit. The PCR product was further purified using the Quant-iT PicoGreen dsDNA Assay Kit. The library was quantified on the Promega QuantiFluor fluorescence quantification system. The pooled library was loaded on Illumina platform (250PE) using a paired-end sequencing protocol (2 × 250 bp).

### 2.6. Bioinformatics Analysis

The sequences were assembled through the FLASH (V1.2.11) according to the overlapping relationship of two terminal sequencing results. Vsearch (v2.3.4) software was used to filter the chimeras to obtain high-quality clean tags. Tags with sequence similarity greater than 97% were defined as an operational taxonomic unit (OTU). The OTU representative sequence was selected. Ribosomal Database Project (RDP) classifier (V2.2) based on NCBI-16s database was used to annotate each OTU representative sequence. The α diversity index (Chao and Simpson index) and microbial composition analysis were performed using QIIME (V1.9.0). The top 30 dominant bacteria genera in each group were selected for later analysis. Principal component analysis and OPLS-DA were performed using SIMCA (14.1) and the R^2^(X), R^2^(Y), Q^2^ of the model was calculated. The differential genera were screened through variable importance in projection (VIP) analysis through permutation test (number of permutations to 200), where genera with VIP value > 1 (*p* < 0.05) were regarded as the differential genus. 

### 2.7. Faecal Metabolomics

100 μg faecal samples were mixed with 1200 μL methanol. The mixture was under ultrasonic treatment for 15 min and then centrifuged at 12,000 rpm for 10 min. Two hundred μL of supernatant was placed in a derivatized sample bottle and blown dry with nitrogen. Fifty μL methoxamine pyridine solution was added and placed in a water bath at 70 °C for 1 h. After the water bath, the mixture was cooled to room temperature, 50 μL derivatization reagent was added and the mixture was placed in a water bath at 70 °C for 1 h. After the water bath, the mixture was cooled to room temperature and 100 μL n-heptane was added. Next, the mixture was vortexed and centrifuged at 12,000 rpm for 15 min. The supernatant was taken for GC-MS analysis. The multivariate analysis of the obtained data was performed in SIMCA (14.1) by PCA and OPLS-DA. The R^2^(X), Q^2^ of the PCA model and the R^2^(X), R^2^(Y), Q^2^ of the OPLS-DA model were calculated. The differential metabolites were screened through variable importance in projection (VIP) analysis through the permutation test (number of permutations to 200), where metabolites with VIP value > 1 (*p* < 0.05) were regarded as the differential metabolites.

### 2.8. Statistical Analysis 

Statistical analysis was performed by GraphPad Prism 8.0. Differences in experimental data were analyzed by one-way ANOVA with SPSS 19.0 and the means were separated using Duncan’s multiple test (* *p* < 0.05, ** *p* < 0.01).

## 3. Results

### 3.1. Body Weight and DAI

**Figure 1:**
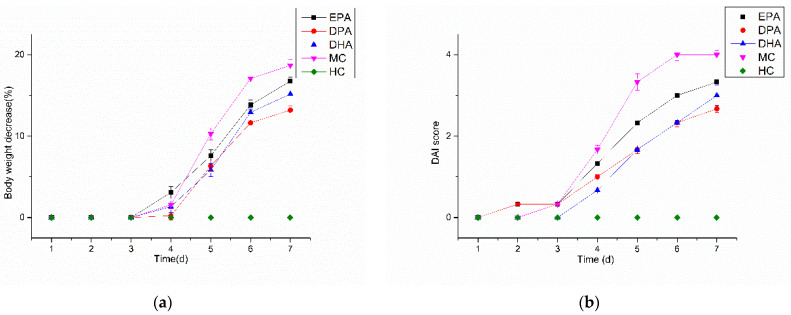
Changes in bodyweight (**a**) and DAI (**b**) score during DSS treatment. Note: EPA, DPA, DHA, MC, HC represent EPA group, DPA group, DHA group, model control group, health control group, respectively.

Changes in the body weight of the mice during the DSS treatment are shown in Figure 1a. The body weight of the mice in the HC group remained stable and there was no body weight loss. For the others, body weight loss began on the fourth day and the maximum was observed on the seventh day. For the MC group, the maximum was 18.69% while the weight loss condition was improved in the EPA (16.76%), DPA (13.20%), and DHA (15.17%) groups. The pathological performance of UC is complex, and body weight change cannot mirror the development of the disease comprehensively. The DAI integrates and quantifies the index of body weight loss, hematochezia, and fecal condition, which is used widely in evaluation of the development of UC. Changes of the DAI score in mice during DSS treatment are shown in Figure 1b. The DAI scores in the HC group kept at 0 during the DSS treatment period. The other four groups increased rapidly after the third day; the DAI scores in the EPA, DPA, DHA, and the MC group increased from 0.33 to 3.33, 0.33 to 2.67, 0 to 3.00, and 0.33 to 4.00, respectively. The results indicated that the body weight decrease and DAI increase were inhibited effectively by the intervention of EPA, DPA, and DHA. Moreover, DPA was the most effective.

### 3.2. Colon Morphology

**Figure 2:**
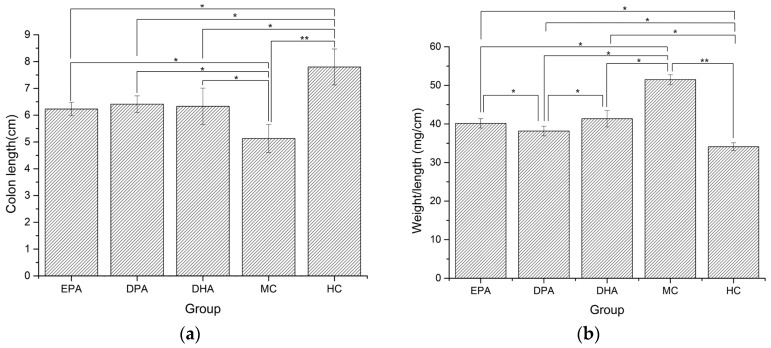
Changes in colon length (**a**) and weight/length ratio (**b**) during the DSS treatment. Note: EPA, DPA, DHA, MC, HC represent EPA group, DPA group, DHA group, model control group, health control group, respectively. * *p* < 0.05, ** *p* < 0.01.

Changes of colon morphology in different groups are shown in Figure 2. The length of colon in the MC group decreased 34.23% and the value of weight/length increased by 50.98%, compared with the HC group. The colon lengths of the EPA, DPA, and DHA groups were 6.23 cm, 6.41 cm, and 6.33 cm, which were 21.14%, 24.95%, and 23.39% longer than that of the MC group (Figure 2a). The weight/length ratios of EPA, DPA, and DHA groups were 40.15 mg/cm, 38.16 mg/cm, and 41.35 mg/cm, which were 22.18%, 22.15%, and 24.55% lower than that of the MC group (Figure 2b). These results suggested that EPA, DPA, and DHA could effectively ameliorate the DSS-induced colon shortening and edema in mice.

### 3.3. Composition and Diversity of the Gut Microbiota

**Figure 3:**
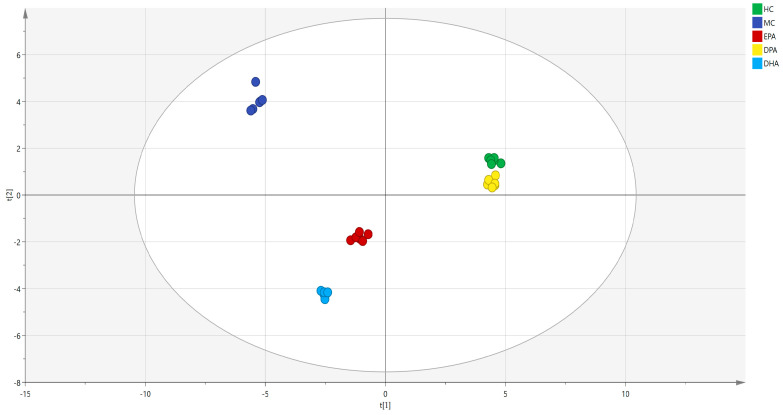
The correlation analysis of the gut microbiota in different groups was evaluated by PCA. Note: t (1) and t (2) represent the corresponding explanation rate of principal component 1 (56.2%) and principal component 2 (31.4%); EPA, DPA, DHA, MC, HC represent EPA group, DPA group, DHA group, model control group, health control group, respectively.

The Venn diagram of the gut microbiota in the five groups is shown in Figure S1. The distribution of microorganisms in each group was relatively dispersed, of which 68 OTU were detected in all groups. There were 430 OTU detected only in the HC group. The numbers of these in the MC group, EPA group, DPA group, and DHA group were 199, 309, 361, and 222. This result indicated that DSS treatment destroyed the diversity of intestinal flora in mice. The correlation analysis of the gut microbiota in different groups at genera level was conducted by PCA (R^2^(X) = 0.967, Q^2^ = 0.945) and the results are shown in Figure 3. The distance between the representative points of the MC group and the HC group was the farthest, which indicated that DSS induction seriously disturbed the composition of intestinal flora in mice. The distance between EPA, DPA, DHA group and the HC group was shorter than that of the MC group and the HC group. Moreover, the sample points of the DPA group were the nearest to the HC group. All these results indicated that supplementation of EPA, DPA, and DHA restored the disorder of intestinal flora caused by DSS, and DPA was the most efficient. 

**Figure 4:**
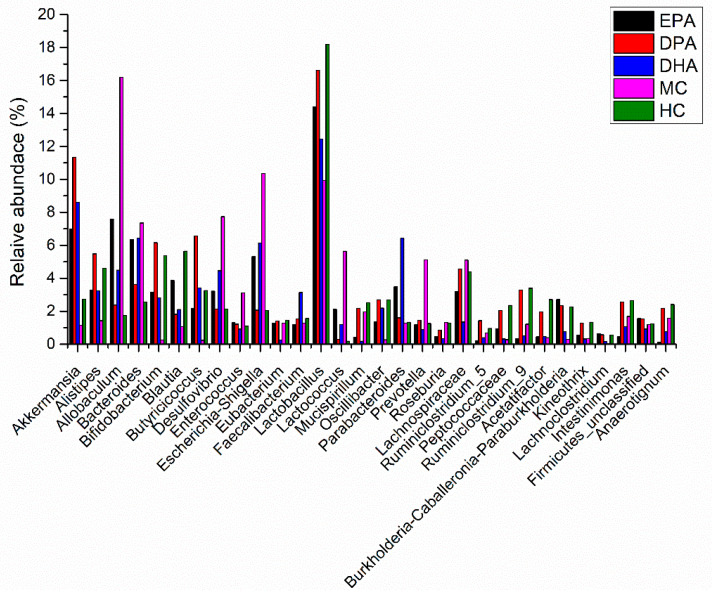
The abundance changes of the gut microbiota in the different groups at genera level. Note: EPA, DPA, DHA, MC, HC represent EPA group, DPA group, DHA group, model control group, health control group, respectively.

The species classification histogram of each group at the phylum level is shown in Figure S2. The flora composition in each group was similar, but the proportion was different. The predominant microbes were *Firmicutes*, *Bacteroidetes*, *Proteobacteria*, *Actinobacteria,* and *Deferribacteres*. Many microbes of *Firmicutes* promote the resolving of inflammation via endogenous anti-inflammatory mediators. Besides, the ratio of *Firmicutes* and *Bacteroidetes* is positively correlated with obesity and inflammation degree. The relative abundance of *Firmicutes* and *Bacteroidetes* in HC group was 51.75%, and 40.16%. The ratio of *Firmicutes* and *Bacteroidetes* (F/B) was 1.29. The DSS treatment decreased the relative abundance of *Firmicutes* (39.15%) and *Bacteroidetes* (20.57%) while it increased the ratio of F/B (1.90). The decline trend of *Firmicutes* and *Bacteroidetes* was inhibited in the EPA, DPA, and DHA groups, as did the increase trend of F/B ratio. Many pathogens, such as *E. coli, Salmonella, Vibrio cholerae,* and *Helicobacter pylori*, belong to the phylum of *Proteobacteria*. These bacteria usually destroy the permeability of the intestines and exacerbate the development of UC. On the contrary, *Actinobacteria* is another phylum of bacteria including many probiotics. They regulate the immune response and enhance innate defense ability. The relative abundance of *Proteobacteria* in the HC group was 2.39%, and it added up to 17.58% in MC group. Supplementation of EPA, DPA, and DHA inhibited the increase trend. The relative abundance of *Actinobacteria* presented the opposite trend. Therefore, DSS treatment decreased the relative abundance of *Firmicutes*, *Bacteroidetes,* and *Actinobacteria*, while it increased the relative abundance of *Proteobacteria* and the ratio of F/B. Supplementation of EPA, DPA, and DHA improved these conditions. The changes in the relative abundance of the gut microbiota in the different groups at genera level are shown in Figure 4. Compared with the HC group, the abundance of the MC group changed significantly. Major genera with increased trend included *Allobaculum, Bacteroides, Desulfovibrio, Enterococcus, Esherichia/Shigella,* and *Prevotella*. Major genera with decreased trend included *Akkermansia, Alistipes, Bifidobacterium, Blautia, Butyricicoccus, Lactobacillus,* and *Ruminiclostridium.* Intervention with EPA, DPA, and DHA modified this disorder.

The alpha diversity analysis results of each group are shown in Table 1. The coverage values of the five groups were close to 1, indicating that the sequencing results could reflect the real situation of the samples. Compared with the HC group, the ACE, Shannon and Chao index of the MC group decreased significantly (*p* < 0.05), indicating that the community richness and diversity of the microbes in the mice colon were destroyed by DSS treatment. This condition was improved in the EPA, DPA, and DHA groups. Moreover, the condition in the DPA group was more close to the healthy controls indicating that DPA might be more effective in the regulation of the gut microbiota.

### 3.4. The Screening of the Differential Bacterial Genus

**Figure 5:**
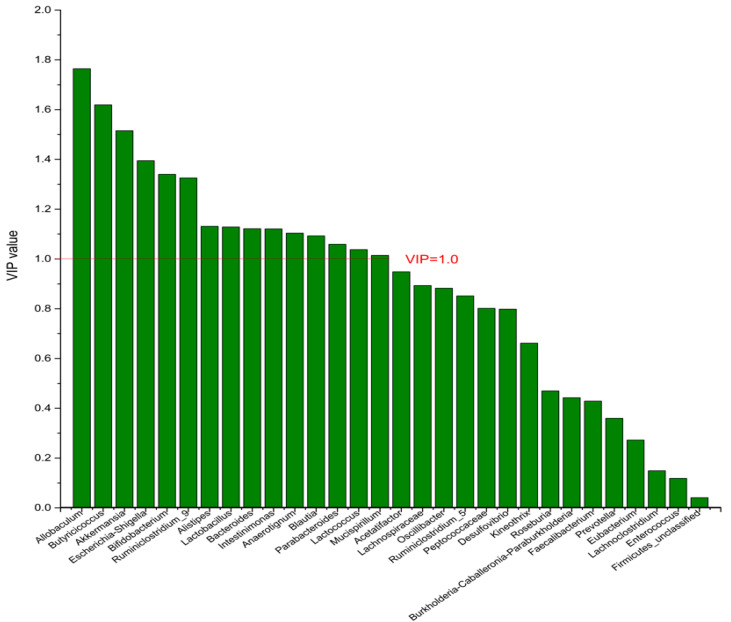
The VIP plot of the differential bacterial genera in DPA group vs. EPA group.

**Figure 6:**
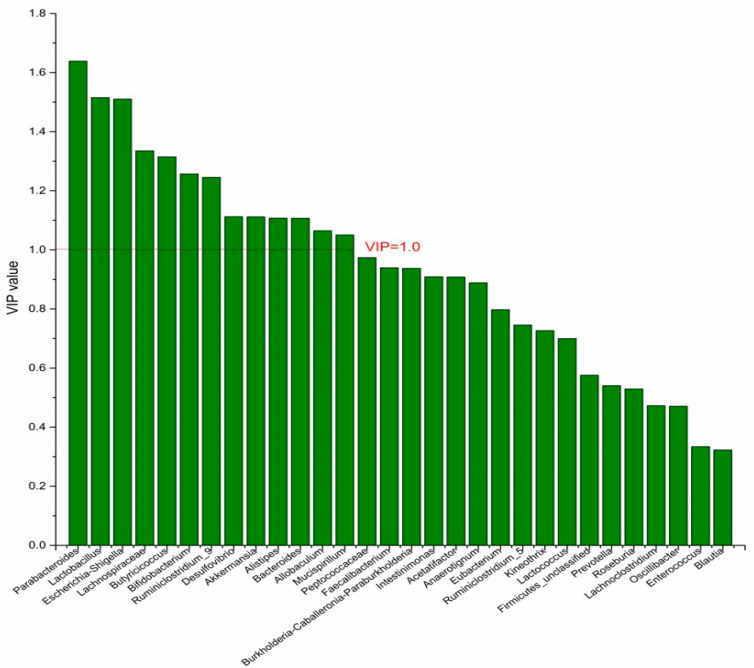
The VIP plot of the differential bacterial genera in DPA group vs. DHA group.

**Figure 7:**
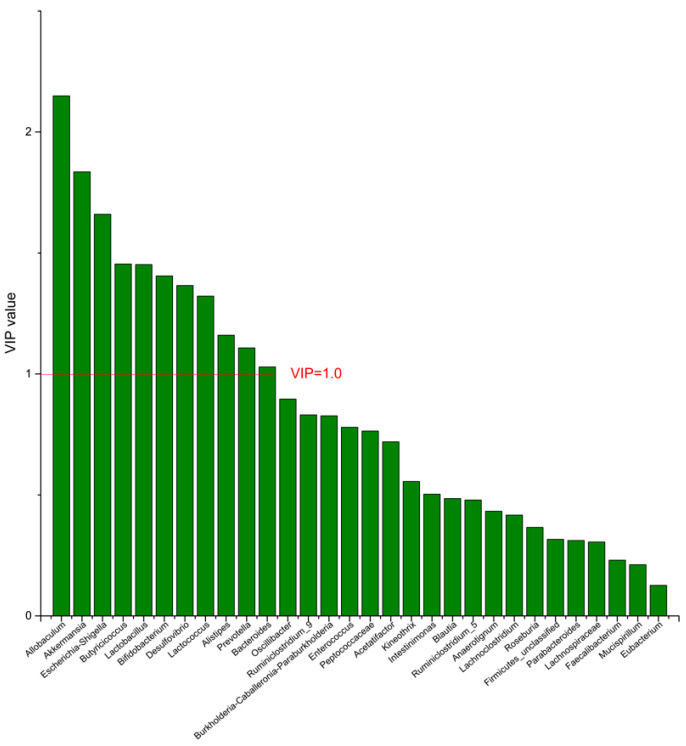
The VIP plot of the differential bacterial genera in DPA group vs. MC group.

**Figure 8:**
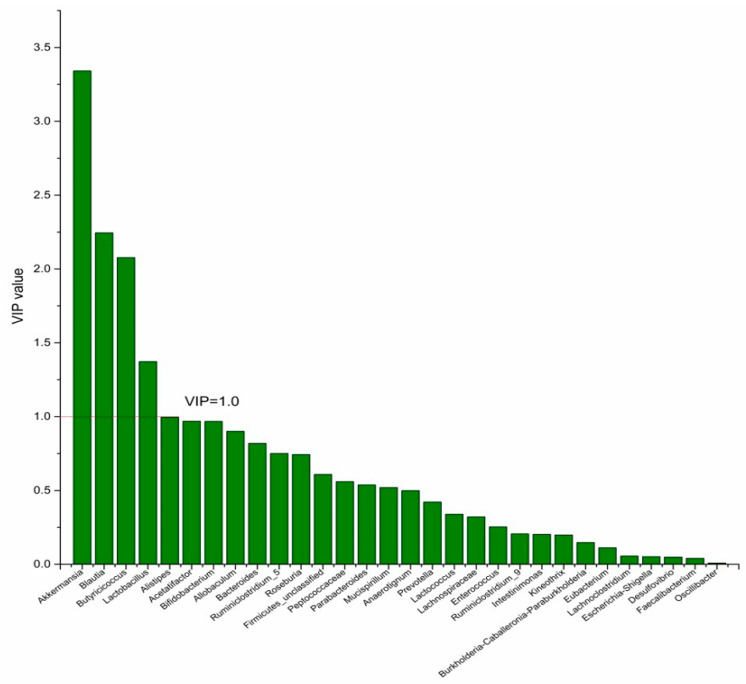
The VIP plot of the differential bacterial genera in DPA group vs. HC group.

To identify the differential bacterial genera in responding to DPA supplementation, OPLS-DA was performed. The R^2^(X), R^2^(Y), Q^2^ of the model of DPA vs. EPA was 0.949, 0.999, 0.998, respectively. The R^2^(X), R^2^(Y), Q^2^ of the model of DPA vs. DHA was 0.937, 0.999, 0.997. These values of the mode of DPA vs. MC, DPA vs. HC were 0.955, 1, 0.998 and 0.835, 0.995, 0.987. The fitness of the models was good due to the high values of R^2^(X), R^2^(Y), and Q^2^. Permutation tests (numbers of permutations to 200) of these models were conducted and the VIP values of the genus were calculated (Tables S1 and S2). Genera with VIP > 1.0 (*p* < 0.05) were selected as the differential genus. The results are shown in Figure 5, Figure 6, Figure 7 and Figure 8. There were 15 differential bacterial genera between the DPA group and the EPA group, which were *Allobaculum*, *Butyricicoccus*, *Akkermansia*, *Escherichia-Shigella*, *Bifidobacterium*, *Ruminiclostridium_9*, *Alistipes*, *Lactobacillus*, *Bacteroides*, *Intestinimonas*, *Anaerotignum*, *Blautia*, *Parabacteroides*, *Lactococcus*, and *Mucispirillum*. Thirteen differential bacterial genera between the DPA group and the DHA group were screened. They were *Parabacteroides*, *Lactobacillus*, *Escherichia-Shigella*, *Lachnospiraceae*, *Butyricicoccus*, *Bifidobacterium*, *Ruminiclostridium_9 Desulfovibrio*, *Akkermansia*, *Alistipes*, *Bacteroides*, *Allobaculum*, and *Mucispirillum*. Eleven differential bacterial genera between the DPA group and the MC group were screened. They were *Allobaculum*, *Akkermansia*, *Escherichia-Shigella*, *Butyricicoccus*, *Lactobacillus*, *Bifidobacterium*, *Desulfovibrio*, *Lactococcus*, *Alistipes*, *Prevotella*, and *Bacteroides*. Five differential bacterial genera between the DPA group and the HC group were screened. They were *Akkermansia*, *Blautia*, *Butyricicoccus*, *Lactobacillus*, and *Alistipes*. Among the entire differential bacterial genera, *Akkermansia*, *Alistipes*, *Butyricicoccus*, and *Alistipes* were differential bacterial genera in all the compare groups. Therefore, they selected as the specific bacterial genera in responding to DPA supplementation. 

### 3.5. The Screening of the Differential Metabolites

**Figure 9:**
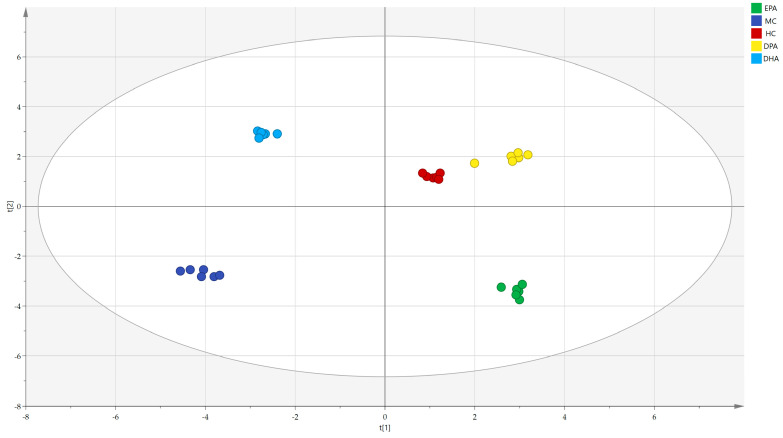
The correlation analysis of the metabolites in different groups evaluated by PCA. Note: t (1)and t (2) represent the corresponding explanation rate of principal component 1 (46.1%) and principal component 2 (39.4%); EPA, DPA, DHA, MC, HC represent EPA group, DPA group, DHA group, model control group, health control group, respectively.

The correlation analysis of the metabolites in different groups was conducted by PCA (R^2^(X) = 0.929, Q^2^ = 0.813) and the results are shown in Figure 9. The distance between the representative points of the MC group and the HC group was the farthest, while that of the DPA group and HC group was the nearest, indicating that supplementation of EPA, DPA, and DHA could restore the metabolic disorders of the gut microbiota and DPA was the most efficient.

**Figure 10:**
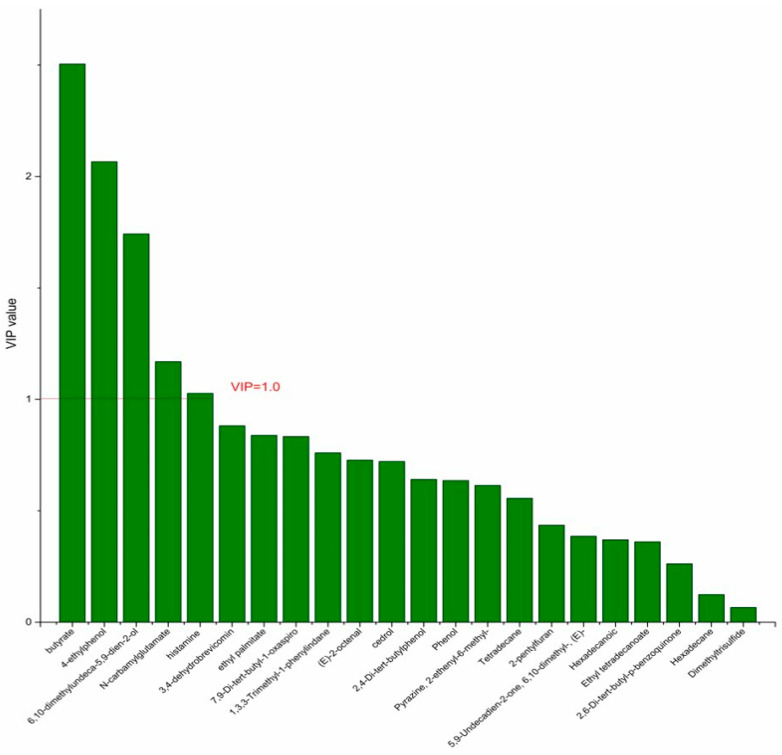
The VIP plot of the differential bacterial metabolites in DPA group vs. EPA group.

**Figure 11:**
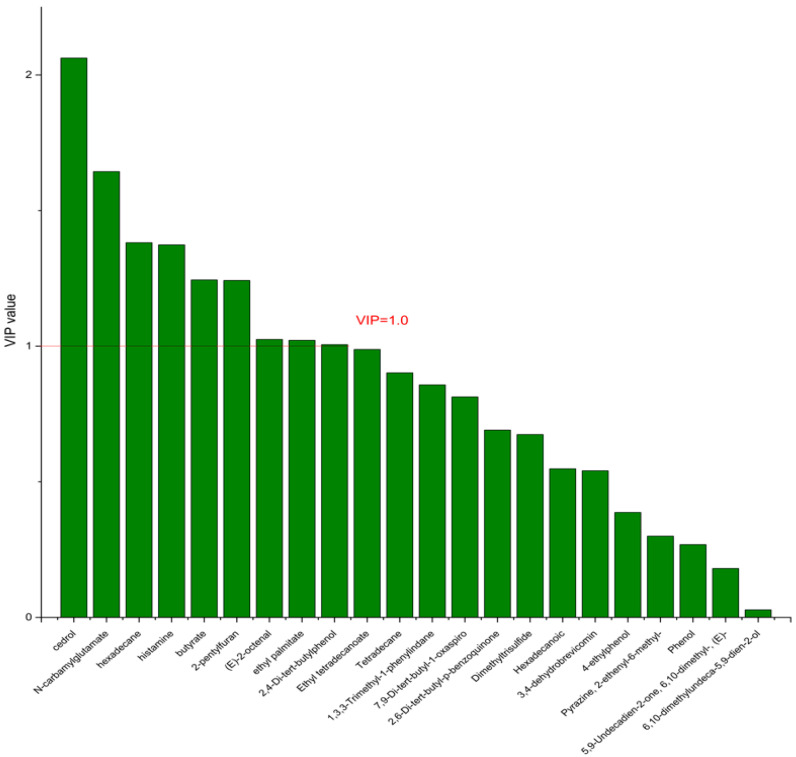
The VIP plot of the differential bacterial metabolites in DPA group vs. DHA group.

**Figure 12:**
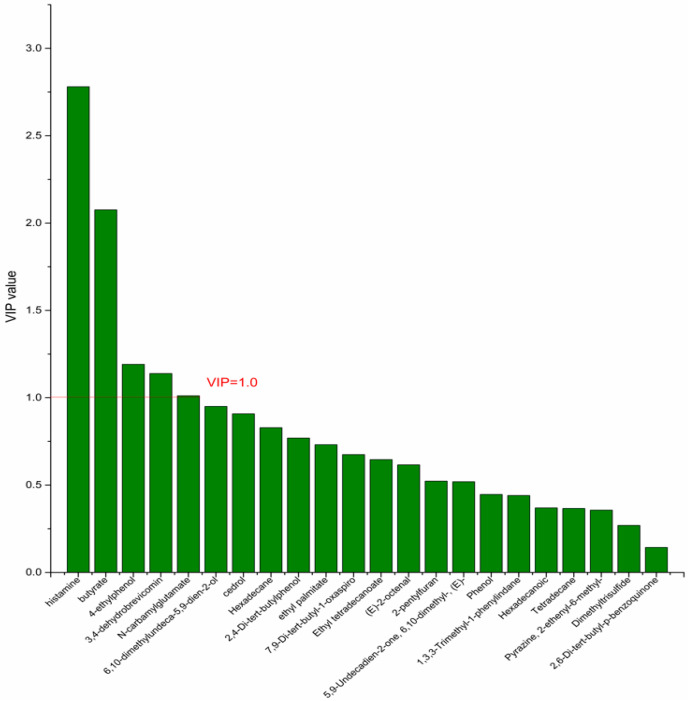
The VIP plot of the differential bacterial metabolites in DPA group vs. MC group.

**Figure 13:**
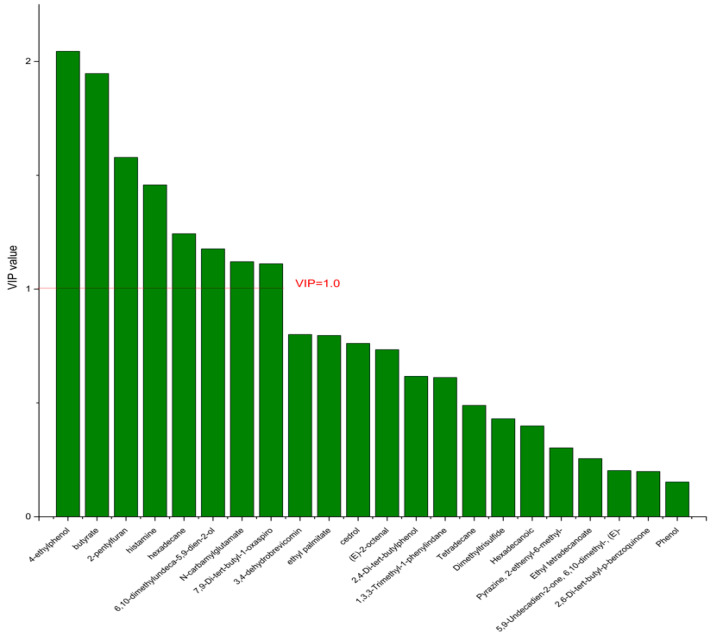
The VIP plot of the differential bacterial metabolites in DPA group vs. HC group.

On the basis of the PCA results, the supervised OPLS-DA was used to screen the differential metabolites. The R^2^(X), R^2^(Y), Q^2^ of the model of DPA vs. EPA was 0.975, 0.999, 0.998, respectively. The R^2^(X), R^2^(Y), Q^2^ of the model of DPA vs. DHA was 0.95, 0.997, 0.995. These values of the mode of DPA vs. MC, DPA vs. HC were 0.968, 0.999, 0.998 and 0.949, 0.999, 0.997. The fitness of the models was good due to the high values of R^2^(X), R^2^(Y), and Q^2^. The permutation tests (numbers of permutations to 200) of these models were conducted and the VIP values of the metabolites were calculated (Tables S3 and S4). Metabolites with VIP > 1.0 (*p* < 0.05) were selected as the differential metabolites. The results are shown in Figure 10, Figure 11, Figure 12 and Figure 13. There were five differential metabolites between the DPA group and the EPA group, which were butyrate, 4-ethylphenol, 6,10-dimethylundeca-5,9-dien-2-ol, *N*-carbamylglutamate, histamine. Nine differential metabolites between the DPA group and the DHA group were selected. They were cedrol, *N*-carbamylglutamate, hexadecane, histamine, butyrate, 2-pentylfuran, (E)-2-octenal, ethyl palmitate, 2,4-Di-tert-butylphenol. Five differential metabolites between the DPA group and the MC group were selected. They were histamine, butyrate, 4-ethylphenol, 3,4-dehydrobrevicomin, *N*-carbamylglutamate. Eight differential metabolites between the DPA group and the HC group were selected. They were 4-ethylphenol, butyrate, 2-pentylfuran, histamine, hexadecane, 6,10-dimethylundeca-5,9-dien-2-ol, *N*-carbamylglutamate, 7,9-Di-tert-butyl-1-oxaspiro[4.5]deca-6,9-diene-2,8-dion. Among the entire differential metabolites, butyrate, *N*-carbamylglutamate, and histamine were differential metabolites in all the compare groups. Therefore, they were selected as the specific metabolites in responding to DPA supplementation.

## 4. Discussion

Ulcerative colitis, as an incurable disease in modern society, has the characteristics of long courses and easy recurrence. The side effects of traditional drugs lead people to turn their attention to dietary intervention [15]. In this paper, we found that prior intervention of DPA was effective in relieving the symptoms of UC in mice, such as body weight decrease, DAI score, and colon morphology. Moreover, DPA supplementation significantly improved the gut microbial dysbiosis and increased microbial diversity. *Akkermansia*, *Alistipes*, *Butyricicoccus*, *Lactobacillus* were selected as the specific bacterial genera in responding to DPA supplementation. Besides, DPA supplementation also improved the fecal metabolite dysbiosis in the UC mice. The PCA results of the metabolites in different groups indicated that the fecal metabolite profile of DPA was the most similar to that of the HC group. Key metabolites such as butyrate, NCG, and histamine were screened as the differential metabolites for DPA supplementation. All these results confirmed the hypotheses that the mechanism by which DPA intervened in UC also involved its regulation of the gut microbiota and its metabolites preliminarily. For DPA, the major source is obviously seafood, including fish from the *Clupeidae* family that gave the n-3 DPA its common name: clupanodonic acid. Seal meat and fats appear to be the richest in n-3 DPA, containing 5.6% of n-3 DPA [16]. Salmon contains 393 mg of n-3 DPA per 100 g serving, Atlantic mackerel 200 mg, and other oily fish between 100 and 200 mg [17]. Beef liver and lamb are the richest land-based sources of n-3 DPA, at up to 140 mg of n-3 DPA/100 g [18]. Recently, an Australian team induced the aerobic long chain PUFA biosynthesis pathway into *Brassica juncea* and a transgenic *B. juncea* with the production of 12% DPA in its seed oil was selected [19]. This work will facilitate subsequent studies and production of DPA. Last but not least, research into the multiple interplays between specific bacterial genera and differential metabolites should be conducted in future to explain the mechanism systematically.

Gut microbiota refers to microorganisms that have long settled on the surface of the intestinal mucosa or in the intestinal cavity, and constitute the intestinal biological barrier. In recent years, with the deep realization of gut microbiota, it has been found that the state of gut microbiota is closely related to the development of UC [20,21]. Tautog et al. [22] raised genetically modified mice under sterile conditions and found that enteritis was not developed, indicating that the occurrence of enteritis requires the participation of microorganisms. Another study [23] found a significant decrease in the diversity of gut microbiota in UC patients, as well as significant changes in composition, such as a significant decrease in the abundance of *Bacteroides* and *Firmicutes*, and an increase in the abundance of *Proteobacteria*, indicating the important role of gut microorganisms in UC. All these studies have shown the important role of gut microbes in UC. In this study, we found that the DSS-induce significantly decreased the abundance of *Akkermansia, Alistipes, Bifidobacterium, Blautia, Butyricicoccus, Lactobacillus,* and *Ruminiclostridium* while increased the abundance of *Allobaculum, Bacteroides, Desulfovibrio, Enterococcus, Esherichia/Shigella,* and *Prevotella*. This disorder was improved in the mice supplemented with EPA, DPA, and DHA. Moreover, DPA was more effective in the regulation of *Akkermansia*, *Alistipes*, *Butyricicoccus*, and *Lactobacillus*.

*Akkermansia*, *Alistipes*, *Butyricicoccus*, and *Lactobacillus* are important members of the gut microbiota. These microbes play positive roles in maintaining the healthy state of the gut. *Akkermansia* is one kind of intestinal bacteria using gastrointestinal mucous protein for growth. It is closely related to the process of immune response and lipid metabolism. Recent studies have shown that *Akkermansia* has unique effects in restoring intestinal barrier function and promoting the production of short-chain fatty acids [24,25]. *Alistipes* is a genus related to irritable bowel syndrome, fatigue, and depression. Studies have shown that the abundance of this genus is negatively correlated with the expression of pro-inflammatory factors in the host, indicating that it plays an important role in regulating inflammation. In addition, this genus is also closely related to the activation of the immune system [26]. *Butyricicoccus* is one of the important members of *Firmicutes*. It promotes the production of butyric acid and is considered as a promising probiotic for the treatment of UC. Butyric acid could be easily absorbed by colon cells and maintain intestinal health by promoting cell differentiation, cell cycle arrest, and the transmission of apoptotic colon cells [27]. *Lactobacillus* is a gram-negative bacterium that is widely distributed in nature. Studies have proved that it is effective in the production of organic acids and it has been helpful in alleviating DSS-induced UC in mice [28]. Although many studies indicated that n-3PUFA supplementation regulates the gut microbiota, given that the absorption of fatty acid mostly occurs in the small intestine, DPA may not directly affect the gut microbiota and may act through its circulation or other intermediates (e.g., resolvins). Studies about the circulation and metabolism of DPA in vivo need to be carried out in the future.

In this study, we also found that DPA significantly affects the content of some fecal metabolites. Butyrate, NCG, and histamine were screened as differential metabolites. Butyrate is the preferred energy-yielding substrate for colonic cells. It plays an important role in maintaining colonic health by stimulating cell differentiation, cell cycle arrest, and the transmission of apoptotic colonic cells [29]. Studies have indicated a reduction in levels of fecal butyrate in patients with UC to varying degrees. Subsequently, some studies have indicated that butyrate enemas alleviate UC clinical symptoms [30,31,32]. The results of this study showed that DPA had an obvious effect on butyrate content, which was significantly consistent with its effect on *Butyricicoccus* and *Akkermansia*. However, it is well known that the main pathway for the production of short-chain fatty acids is the fermentation of dietary fiber by specific bacterial groups. From the results presented in this article, we speculate that the existence of DPA might promote the β-oxidation process of fatty acids and indirectly promote the production of short-chain fatty acids such as butyric acid, but more in-depth and detailed research needs to be carried out.

*N*-carbamylglutamate (NCG) is an analogue of *N*-acetylglutamic acid (NAG) that can act similarly to NAG in animals and participate in the urea cycle. *N*-carbamylglutamate has a long half-life and stable metabolism, and has a wide range of biological functions [33]. Studies have shown that NCG acts as a metabolic activator to participate in the activation of dihydropyrrolid-5-carboxylate synthetase (P5CS) and carbamoyl phosphate synthetase I (CPS-I), promote the synthesis of citroline from glutamine or proline, and then promote the synthesis of arginine, which play an important role in regulating nutrient metabolism and immune response [34]. An animal experiment showed that that NCG enhanced intestinal growth and the heat shock protein expression by modification of the arginine metabolism [35]. Another study indicated that NCG alleviates UC through reducing oxidative stress in the gut [36].

Histamine is a nitrogenous organic compound and it is produced by histidine under the action of decarboxylase. Many tissues, especially the mast cells of the skin, lungs, and intestinal mucosa, contain a large amount of histamine, which can be released when the tissue is damaged or when inflammation or allergic reactions occur. Histamine and its metabolites are closely related to the occurrence and development of UC [37,38,39]. An in vivo study showed that the expression of histamine and histamine 4 receptor significantly increased in mice with UC [40]. Another clinical study showed that the content of histamine and its related metabolite, *N*- *N*-methylhistamine, increased significantly in the urine of UC patients [41]. One study involved mechanisms indicating that histamine exacerbated the inflammatory response by activating inflammatory mediator receptors in UC mice [42]. In this study, we found that the histamine content in the MC group increased significantly, compared with the HC group, and DPA supplementation significantly inhibited this increase. Since decarboxylase is produced mostly by microorganism, the regulation effect of DPA on the gut microbiota might be the underlying mechanism.

## 5. Conclusions

In conclusion, we reported that DPA could effectively alleviate the symptoms of UC in mice induced by DSS. The mechanism underlying this effect might involve its effect on the diversity and composition of gut microbiota and their metabolites. More research needs to be carried out to elucidate the mechanism precisely.

## Figures and Tables

**Table 1:** Alpha diversity of samples for all groups (* *p* < 0.05).

	ACE	Shanno	Simpson	Chao	Coverage
EPA	570.15 ± 25.41 *	6.23 ± 0.15 *	0.85 ± 0.02	573.39 ± 21.25 *	0.9968 ± 0.0010
DPA	615.31 ± 31.64 *	6.95 ± 0.21 *	0.86 ± 0.01	613.88 ± 22.35 *	0.9925 ± 0.0021
DHA	484.26 ± 18.26 *	5.93 ± 0.11 *	0.86 ± 0.03	486.00 ± 19.58 *	0.9915 ± 0.0011
MC	425.10 ± 20.35 *	4.53 ± 0.25 *	0.90 ± 0.02 *	431.38 ± 23.14 *	0.9975 ± 0.0013
HC	628.75 ± 25.67 *	7.15 ± 0.16 *	0.88 ± 0.01 *	631.05 ± 25.44 *	0.9924 ± 0.0027

Note: EPA, DPA, DHA, MC, HC represent EPA group, DPA group, DHA group, model control group, health control group, respectively.

## Data Availability

Data are available upon request.

## Supplementary Materials

The following supporting information can be downloaded at: https://www.mdpi.com/article/10.3390/nu14194204/s1, Figure S1: The Venn diagram of the gut microbiota in the different groups. Figure S2: The abundance changes of the gut microbiota in the different groups at phyla level. Table S1: The VIP value of the genera after OPLS-DA in Figure 5 and Figure 6. Table S2: The VIP value of the genera after OPLS-DA in Figure 7 and Figure 8. Table S3: The VIP value of the metabolites after OPLS-DA in Figure 10 and Figure 11. Table S4: The VIP value of the metabolites after OPLS-DA in Figure 12 and Figure 13.
