# Supplementary material for: Docosapentaenoic Acid (DPA, 22:5n-3) Alleviates Ulcerative Colitis via Modification of Gut Microbiota and Their Metabolism
Source: Nutrients. 2022 Oct 9;14(19):4204. doi: 10.3390/nu14194204 (PMC9570819; doi:10.3390/nu14194204)
Supplement: Supplementary file 1 [file nutrients-14-04204-s001.zip › nutrients-1942343-supplementary.pdf]

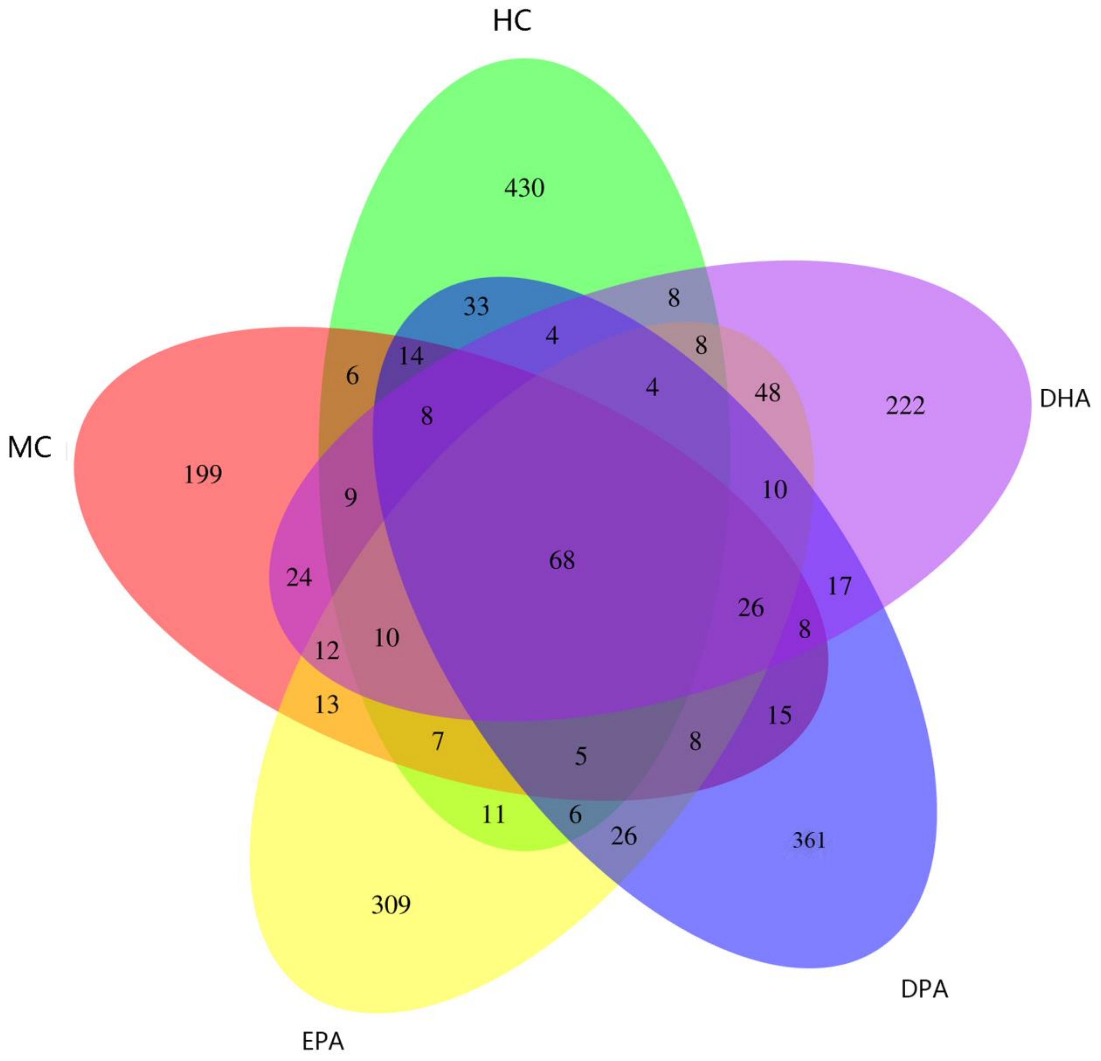

Figure S1. The Venn diagram of the gut microbiota in the different groups. Note: EPA, DPA, DHA, MC, HC represented EPA group, DPA group, DHA group, model control group, health control group respectively.

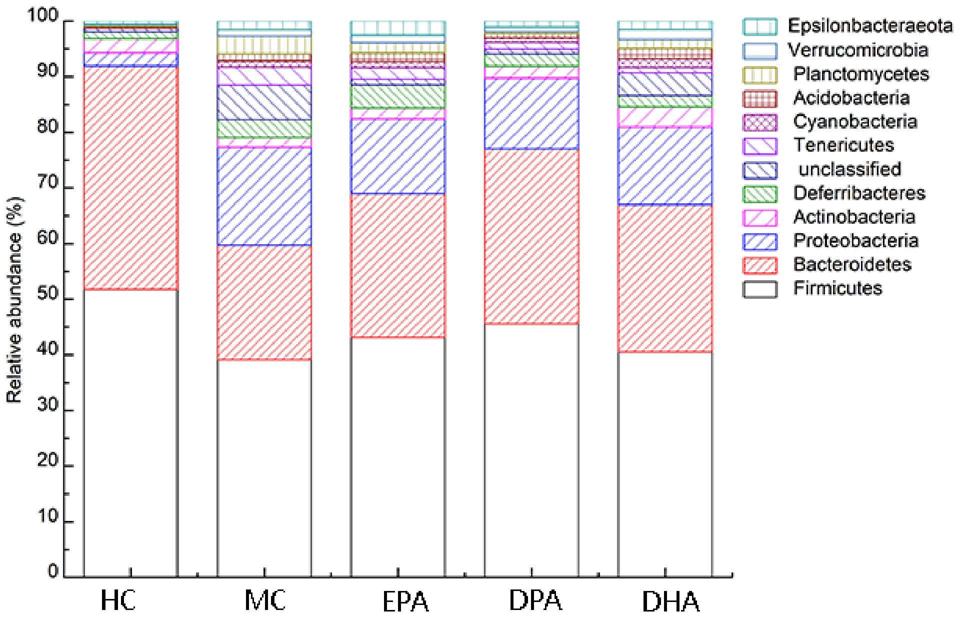

Figure S2. The abundance changes of the gut microbiota in the different groups at phyla level. Note: EPA, DPA, DHA, MC, HC represented EPA group, DPA group, DHA group, model control group, health control group respectively.

Table S1 The VIP value of the genera after OPLS-DA in Figure 5 and Figure 6

Figure 5		Figure 6	
Genera	VIP value	Genera	VIP value
Allobaculum	1.76	*Parabacteroides*	1.64
Butyricicoccus	1.62	*Lactobacillus*	1.52
Akkermansia	1.52	*Escherichia-Shigella*	1.51
Escherichia-Shigella	1.39	*Lachnospiraceae*	1.34
Bifidobacterium	1.34	*Butyricicoccus*	1.31
Ruminiclostridium_9	1.33	*Bifidobacterium*	1.26
Alistipes	1.13	*Ruminiclostridium_9*	1.25
Lactobacillus	1.13	*Desulfovibrio*	1.11
Bacteroides	1.12	*Akkermansia*	1.11
Intestinimonas	1.12	*Alistipes*	1.11
Anaerotignum	1.1	*Bacteroides*	1.11
Blautia	1.09	*Allobaculum*	1.06
Parabacteroides	1.06	*Mucispirillum*	1.05
Lactococcus	1.04	*Peptococcaceae*	0.97
Mucispirillum	1.01	*Faecalibacterium*	0.94
Acetatifactor	0.95	*Burkholderia-Caballeronia-Paraburkholderia*	0.94
Lachnospiraceae	0.89	*Intestinimonas*	0.91
Oscillibacter	0.88	*Acetatifactor*	0.91
Ruminiclostridium_5	0.85	*Anaerotignum*	0.89

Peptococcaceae	0.8	*Eubacterium*	0.8
Desulfovibrio	0.8	*Ruminiclostridium_5*	0.75
Kineothrix	0.66	*Kineothrix*	0.73
Roseburia	0.47	*Lactococcus*	0.7
Burkholderia-Caballeronia-Paraburkholderia	0.44	*Firmicutes_unclassified*	0.58
Faecalibacterium	0.43	*Prevotella*	0.54
Prevotella	0.36	*Roseburia*	0.53
Eubacterium	0.27	*Lachnoclostridium*	0.47
Lachnoclostridium	0.15	*Oscillibacter*	0.47
Enterococcus	0.12	*Enterococcus*	0.33
Firmicutes_unclassified	0.04	*Blautia*	0.32

Table S2 The VIP value of the genera after OPLS-DA in Figure 7 and Figure 8

Figure 7		Figure 8	
genera	VIP value	genera	VIP value
Allobaculum	2.15	*Akkermansia*	3.34
Akkermansia	1.84	*Blautia*	2.24
Escherichia-Shigella	1.66	*Butyricicoccus*	2.08
Butyricicoccus	1.45	*Lactobacillus*	1.37
Lactobacillus	1.45	*Alistipes*	1
Bifidobacterium	1.41	*Acetatifactor*	0.97
Desulfovibrio	1.37	*Bifidobacterium*	0.97
Lactococcus	1.32	*Allobaculum*	0.9
Alistipes	1.16	*Bacteroides*	0.82
Prevotella	1.11	*Ruminiclostridium_5*	0.75
Bacteroides	1.03	*Roseburia*	0.74
Oscillibacter	0.9	*Firmicutes_unclassified*	0.61
Ruminiclostridium_9	0.83	*Peptococcaceae*	0.56
Burkholderia-Caballeronia-Paraburkholderia	0.83	*Parabacteroides*	0.54
Enterococcus	0.78	*Mucispirillum*	0.52
Peptococcaceae	0.76	*Anaerotignum*	0.5
Acetatifactor	0.72	*Prevotella*	0.42
Kineothrix	0.56	*Lactococcus*	0.34
Intestinimonas	0.5	*Lachnospiraceae*	0.32
Blautia	0.48	*Enterococcus*	0.25
Ruminiclostridium_5	0.48	*Ruminiclostridium_9*	0.21
Anaerotignum	0.43	*Intestinimonas*	0.2
Lachnoclostridium	0.42	*Kineothrix*	0.2
Roseburia	0.37	*Burkholderia-Caballeronia-Paraburkholderia*	0.15
Firmicutes_unclassified	0.32	*Eubacterium*	0.11

Parabacteroides	0.31	*Lachnoclostridium*	0.06
Lachnospiraceae	0.31	*Escherichia-Shigella*	0.05
Faecalibacterium	0.23	*Desulfovibrio*	0.05
Mucispirillum	0.21	*Faecalibacterium*	0.04
Eubacterium	0.13	*Oscillibacter*	0.01

Table S3 The VIP value of the metabolites after OPLS-DA in Figure 10 and Figure 11

Figure 10		Figure 11	
Metabolites	VIP value	Metabolites	VIP value
butyrate	2.5	cedrol	2.06
4-ethylphenol	2.07	N-carbamylglutamate	1.64
6,10-dimethylundeca-5,9-dien-2-ol	1.74	hexadecane	1.38
N-carbamylglutamate	1.17	histamine	1.37
histamine	1.03	butyrate	1.24
3,4-dehydrobrevicomin	0.88	2-pentylfuran	1.24
ethyl palmitate	0.84	(E)-2-octenal	1.02
7,9-Di-tert-butyl-1-oxaspiro	0.83	ethyl palmitate	1.02
1,3,3-Trimethyl-1-phenylindane	0.76	2,4-Di-tert-butylphenol	1.01
(E)-2-octenal	0.73	Ethyl tetradecanoate	0.99
cedrol	0.72	Tetradecane	0.9
2,4-Di-tert-butylphenol	0.64	1,3,3-Trimethyl-1-phenylindane	0.86
Phenol	0.64	7,9-Di-tert-butyl-1-oxaspiro	0.81
Pyrazine, 2-ethenyl-6-methyl-	0.61	2,6-Di-tert-butyl-p-benzoquinone	0.69
Tetradecane	0.56	Dimethyltrisulfide	0.67
2-pentylfuran	0.44	Hexadecanoic	0.55
5,9-Undecadien-2-one, 6,10-dimethyl-, (E)-	0.39	3,4-dehydrobrevicomin	0.54
Hexadecanoic	0.37	4-ethylphenol	0.39
Ethyl tetradecanoate	0.36	Pyrazine, 2-ethenyl-6-methyl-	0.3
2,6-Di-tert-butyl-p-benzoquinone	0.26	Phenol	0.27
Hexadecane	0.12	5,9-Undecadien-2-one, 6,10-dimethyl-, (E)-	0.18
Dimethyltrisulfide	0.07	6,10-dimethylundeca-5,9-dien-2-ol	0.03

Table S4 The VIP value of the metabolites after OPLS-DA in Figure 12 and Figure 13

Figure 12		Figure 13	
Metabolites	VIP value	Metabolites	VIP value
histamine	2.78	4-ethylphenol	2.04
butyrate	2.08	butyrate	1.95
4-ethylphenol	1.19	2-pentylfuran	1.58

3,4-dehydrobrevicomin	1.14	histamine	1.46
N-carbamylglutamate	1.01	hexadecane	1.24
6,10-dimethylundeca-5,9-dien-2-ol	0.95	6,10-dimethylundeca-5,9-dien-2-ol	1.18
cedrol	0.91	N-carbamylglutamate	1.12
Hexadecane	0.83	7,9-Di-tert-butyl-1-oxaspiro	1.11
2,4-Di-tert-butylphenol	0.77	3,4-dehydrobrevicomin	0.8
ethyl palmitate	0.73	ethyl palmitate	0.8
7,9-Di-tert-butyl-1-oxaspiro	0.67	cedrol	0.76
Ethyl tetradecanoate	0.65	(E)-2-octenal	0.73
(E)-2-octenal	0.62	2,4-Di-tert-butylphenol	0.62
2-pentylfuran	0.52	1,3,3-Trimethyl-1-phenylindane	0.61
5,9-Undecadien-2-one, 6,10-dimethyl-, (E)-	0.52	Tetradecane	0.49
Phenol	0.45	Dimethyltrisulfide	0.43
1,3,3-Trimethyl-1-phenylindane	0.44	Hexadecanoic	0.4
Hexadecanoic	0.37	Pyrazine, 2-ethenyl-6-methyl-	0.3
Tetradecane	0.37	Ethyl tetradecanoate	0.26
Pyrazine, 2-ethenyl-6-methyl-	0.36	5,9-Undecadien-2-one, 6,10-dimethyl-, (E)-	0.2
Dimethyltrisulfide	0.27	2,6-Di-tert-butyl-p-benzoquinone	0.2
2,6-Di-tert-butyl-p-benzoquinone	0.14	Phenol	0.15
